# Medical students’ satisfaction level with e-learning during the COVID-19 pandemic and its related factors: a systematic review

**DOI:** 10.3352/jeehp.2022.19.37

**Published:** 2022-12-20

**Authors:** Mahbubeh Tabatabaeichehr, Samane Babaei, Mahdieh Dartomi, Peiman Alesheikh, Amir Tabatabaee, Hamed Mortazavi, Zohreh Khoshgoftar

**Affiliations:** 1 Department of Medical Education, ‎Virtual School of Medical Education and Management, Shahid Beheshti University of ‎Medical Sciences, Tehran, Iran; 2 Department of Internal Medicine, ‎School of Medicine, Natural Products and Medicinal Plants Research Center, North ‎Khorasan University of Medical Sciences, Bojnurd, Iran; 3 Department of Nursing, Quchan Branch, Islamic Azad University, ‎Quchan, Iran; 4 Geriatric Care Research Center, Department of Geriatric Nursing, ‎School of Nursing, North Khorasan University of Medical Sciences, Bojnurd, Iran; 5 Department of Medical Education, Virtual School of Medical Education and ‎Management, Shahid Beheshti University of Medical Sciences, Tehran, Iran; Hallym University, Korea

**Keywords:** COVID-19, Distance education, Medical students, Multimedia, Personal satisfaction

## Abstract

**Purpose:**

This review investigated medical students’ satisfaction level with e-learning during the coronavirus disease 2019 (COVID-19) pandemic and its related factors.

**Methods:**

A comprehensive systematic search was performed of international literature databases, including Scopus, PubMed, Web of Science, and Persian databases such as Iranmedex and Scientific Information Database using keywords extracted from Medical Subject Headings such as “Distance learning,” “Distance education,” “Online learning,” “Online education,” and “COVID-19” from the earliest date to July 10, 2022. The quality of the studies included in this review was evaluated using the appraisal tool for cross-sectional studies (AXIS tool).

**Results:**

A total of 15,473 medical science students were enrolled in 24 studies. The level of satisfaction with e-learning during the COVID-19 pandemic among medical science students was 51.8%. Factors such as age, gender, clinical year, experience with e-learning before COVID-19, level of study, adaptation content of course materials, interactivity, understanding of the content, active participation of the instructor in the discussion, multimedia use in teaching sessions, adequate time dedicated to the e-learning, stress perception, and convenience had significant relationships with the satisfaction of medical students with e-learning during the COVID-19 pandemic.

**Conclusion:**

Therefore, due to the inevitability of online education and e-learning, it is suggested that educational managers and policymakers choose the best online education method for medical students by examining various studies in this field to increase their satisfaction with e-learning.

## Introduction

### Background/rationale

The World Health Organization declared in January 2020 the outbreak of the novel severe acute respiratory syndrome coronavirus 2 as an international threat to public health [1]. The infection rate grew rapidly and was declared a global pandemic in March 2020, known as the coronavirus disease 2019 (COVID-19) pandemic [2]. COVID-19 has symptoms similar to those of the common cold, but can cause more severe complications such as bronchitis, pneumonia, or other functional failures, especially in vulnerable people [3]. Although older people are at higher risk for negative outcomes such as mortality, the risks of the COVID-19 pandemic affect everyone [4-10]. Therefore, many countries used public care strategies such as wearing face masks, avoiding gatherings and physical distancing, quarantine, and stay-at-home strategies to control disease transmission [11]. It was impossible to hold classes in such circumstances because doing so would violate disease transmission control strategies [12]. Following this, the education system suffered a severe negative effect. Based on United Nations Educational, Scientific and Cultural Organization estimates, more than 90% of global students could not participate in educational sessions [13]. In such a situation, electronic learning (E-learning) was the only remaining option for universities to continue the professional curriculum and increase students’ educational activities, especially undergraduate medical sciences students [12,14]. E-learning is defined as the use of assistive technologies in teaching online, offline, or in both settings [15]. Part of the dynamism of educational systems in the 21st century depends on e-learning [16]. E-learning can be used as a substitute or supplement for traditional education [17]. By using this learning method, students can save time and continue studying at the university from a long distance [18]. There are different methods for e-learning, such as internet-based learning, computer-based learning, virtual classrooms, and digital collaboration [19]. Previous evidence has identified advantages of e-learning, such as creating better interactions with the instructor and group discussions [1,19,20], the possibility of using multimedia [19], the availability of resources [1,21], and sufficient time to understand the content [19].

Various studies [22-25] have examined medical students’ satisfaction rate with e-learning during the COVID-19 pandemic and its related factors. However, to our knowledge, there is no published study comprehensively reviewing and summarizing the literature regarding medical students’ satisfaction level with e-learning during the COVID-19 pandemic and its related factors.

### Objectives

Given the importance of the subject and the contradictory findings regarding this issue, this systematic review aimed to summarize the evidence regarding medical students’ satisfaction level with e-learning during the COVID-19 pandemic and its related factors. The present study was conducted to answer the following research questions: (1) What was medical science students’ satisfaction level with e-learning during the COVID-19 pandemic? (2) What factors were associated with medical students’ satisfaction with e-learning during the COVID-19 pandemic?

## Methods

### Ethics statement

This was not a study on human or human-origin materials; therefore, neither approval by the institutional review board nor the obtainment of informed consent was required.

### Protocol & registration

This systematic review was described according to the Preferred Reporting Items for Systematic Reviews and Meta-Analysis (PRISMA) guidelines [26]. Also, the present review was not registered in the International Prospective Register of Systematic Reviews (PROSPERO) database due to its website maintenance when we did this research.

### Information sources & search strategy

A comprehensive systematic search was performed of international literature databases, such as Scopus, PubMed, and Web of Science, and Persian electronic databases, such as Iranmedex and Scientific Information Database using keywords extracted from Medical Subject Headings, such as “Distance learning,” “Distance education,” “Online learning,” “Online education,” and “COVID-19” from the earliest date to July 10, 2022. For example, the search strategy in the PubMed/MEDLINE database was ((“Distance learning”) OR (“Distance education”) OR (“Online learning”) OR (“Online education”)) AND ((“Medical science students”) OR (“Medical students”)) AND (“COVID-19”). Keywords were combined using “OR” and “AND” Boolean operators. The Persian equivalents of the keywords were also searched in the Iranian electronic databases. Two researchers separately performed the systematic search. The gray literature, such as expert opinions, conference presentations, theses, research and committee reports, and ongoing research, was not included in this systematic review. Articles in the gray literature are published electronically, but have not been evaluated by a commercial publisher [27].

### Eligibility criteria

In this systematic review, cross-sectional studies focusing on the subject of medical students’ satisfaction with e-learning and related factors are included. Letters to the editor, case reports, conference proceedings, experiments, studies with qualitative designs, and reviews were not included in this review study.

### Study selection

Data management of this systematic review study was done using EndNote 8X software (Clarivate, Philadelphia, PA, USA). The selection of studies in this review was made by 2 researchers separately based on the inclusion and exclusion criteria. At first, the titles, abstracts, the full text of articles, and eliminating duplicate studies were evaluated electronically. Then, to prevent data loss, this process was done manually. The third researcher resolved any contradictions between the 2 researchers in selecting studies. Finally, to prevent data loss, references were checked manually.

### Data collection process, data items, and synthesis of results

The following data were extracted from the articles included in this systematic review: the name of the first author, year of publication, location, sample size, male/female ratio, age, single/married ratio, the field of study of the participants, e-learning modalities, platforms used in e-learning, devices used in e-learning, previous experience of online classes, questionnaire, and key results.

### Risk of bias in individual studies & risk of bias across studies

The quality of the studies included in this systematic review was evaluated using the appraisal tool for cross-sectional studies (AXIS tool). This tool evaluates the quality of the included studies via 20 items with a 2-point Likert scale, including yes (score of 1) and no (score of 0). This tool assesses report quality (7 items), study design quality (7 items), and the possible introduction of biases (6 items). Finally, AXIS rates the quality of studies at 3 levels: high (70% to 100%), fair (60% to 69.9%), and low (0% to 59.9%) [28]. Two researchers extracted the information and evaluated the quality of the studies independently.

### Summary measures

None.

### Additional analyses

Not available.

## Results

### Study selection

**Fig. 1.**
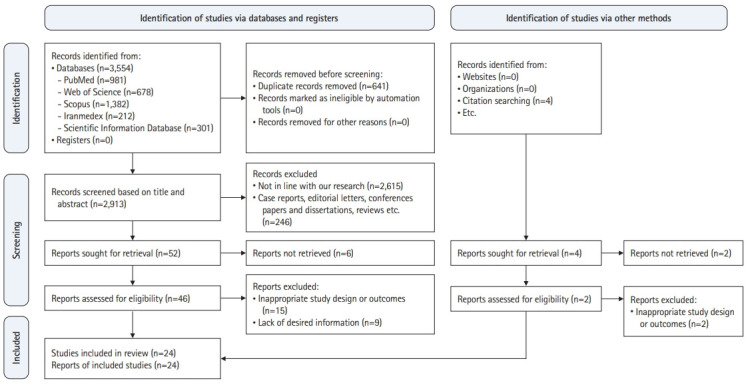
Flow diagram of the study selection process.

As shown in Fig. 1, after an extensive search of electronic databases, 3,554 studies were obtained. Then, 641 duplicate articles were excluded. Of the remaining 2,913 articles, 2,615 studies were excluded because they did not match the research objectives, and 246 articles were excluded due to having a non-cross-sectional design. Of the remaining 52 studies, after a full review of the full-text articles, 15 were excluded from the present systematic review due to an inadequate study design and 9 studies due to a lack of necessary information. Finally, 24 studies remained in this systematic review [1,3,12,19-25,29-42].

### Study characteristics & results of individual studies

As shown in Supplement 1, a total of 15,473 medical science students were enrolled in the 24 studies [1,3,12,19-25,29-42]. The mean±standard deviation age of the participants was 20.84±2.06 years, and 58.23% were women. Of the participants, 73.45% were studying clinical medicine. Of the studies included in this systematic review, 14 studies [19-21,24,25,29-31,33,36-40] reported e-learning modalities, 16 studies [3,19,20,23-25,29,30,32,33,36-40,42] reported platforms used in e-learning, and 10 studies [12,19,22,25,29,32-35,40] reported devices used in e-learning. The studies included in this systematic review were conducted in India (n=5) [1,12,30,39,40], China (n=3) [20,33,34], Saudi Arabia (n=3) [3,21,24], Jordan (n=2) [19,36], Morocco (n=2) [29,37], Ukraine (n=2) [25,42], Indonesia (n=1) [23], Pakistan (n=1) [22], South Korea (n=1) [31], Nepal (n=1) [32], Philippines (n=1) [35], Greece (n=1) [38], and Iran (n=1) [41]. The questionnaires for assessing student satisfaction were created by researchers in 23 studies [1,3,12,19-23,25,29-42]. The reduced version of the Students’ Evaluation of Educational Quality was chosen in 1 study [24].

### Risk of bias within studies & risk of bias across studies

As shown in Supplement 2, of the 24 studies [1,3,12,19-25,29-42] included in this systematic review, 21 studies [1,3,12,19-24,29,31-41] were of high quality, 2 studies [25,30] were of fair quality, and 1 study [42] was of low quality. Ten studies [21,23-25,30,32,33,39,40,42] did not report the selection process of representative participants; 7 studies [25,29,30,34,39,40,42] did not report research limitations; and 2 studies [32,42] did not report funding sources or conflicts of interest.

### Synthesis of results

#### Medical students' satisfaction level with e-learning during the COVID-19 pandemic

The level of satisfaction with e-learning during the COVID-19 pandemic among medical science students was 51.79%.

#### Factors associated with the medical students' satisfaction with e-learning during the COVID-19 pandemic

Among the studies in this systematic review, 9 studies reported factors related to student satisfaction with e-learning [3,19,21,29,33,35-37,41]. The following factors showed a significant relationship with students’ satisfaction with e-learning during the COVID-19 pandemic: gender (n=3) [3,21,41], experience of e-learning before COVID-19 (n=3) [19,36,41], level of study (n=1) [37], adaptation (n=1) [37], content of course materials (n=1) [29], interactivity (n=1) [29], understanding the content (n=1) [29], active participation of the instructor in the discussion (n=1) [19], using multimedia in teaching sessions (n=1) [19], and adequate time dedicated to e-learning (n=1) [19]. The results of the studies showed that there were negative and significant relationships between students’ satisfaction with e-learning and age (n=1) [21], clinical year (n=1) [33], and stress perception (n=1) [35]. There was also a significant positive relationship between students’ satisfaction with e-learning and convenience (n=1) [21] (Supplement 1).

### Additional analyses

Not available.

## Discussion

According to the results of the 24 studies included in this systematic review, almost half of the 15,473 medical students in this study were satisfied with e-learning during the COVID-19 pandemic. Factors such as age, gender, clinical year, experience with e-learning before COVID-19, level of study, adaptation content of course materials, interactivity, understanding of the content, active participation of the instructor in the discussion, using multimedia in teaching sessions, adequate time dedicated to e-learning, stress perception, and convenience had significant relationships with the satisfaction of medical science students with e-learning during the COVID-19 pandemic.

Due to the spread of COVID-19, online training replaced face-to-face training to prevent the spread of the virus [43]. Although online education is known as an effective method for the education of medical science students, the psychological condition of students during the COVID-19 pandemic can affect their satisfaction with online education [33]. The results of this study showed that almost half of the students are satisfied with e-learning. There were differences in the level of satisfaction of medical students with e-learning during the COVID-19 pandemic in the studies included in this systematic review. These differences could have originated from the influence of factors such as age, gender, clinical year, experience with e-learning before COVID-19, level of study, adaptation content of course materials, interactivity, understanding of the content, active participation of the instructor in the discussion, using multimedia in teaching sessions, adequate time dedicated to e-learning, stress perception, and convenience.

A systematic review and meta-analysis conducted by Ahmed et al. showed that 57% of medical students were satisfied with e-learning [14]. A cross-sectional study in Jordan reported that the average score of students’ satisfaction with e-learning was low during the outbreak of COVID-19. It also showed that factors such as level of education, university type, and marital status had a significant relationship with the level of student satisfaction with e-learning [44]. The results of another cross-sectional study in Turkey on the satisfaction of students at different levels of education with e-learning during the COVID-19 pandemic showed that student satisfaction was at a moderate level. That study reported that the level of satisfaction of female students was significantly different from that of male students; students of engineering and social sciences were more satisfied than students of medical and health sciences, and the satisfaction of postgraduate students was significantly different from that of undergraduate and associate-degree students [45]. Another study investigated the level of physician satisfaction with online education during the COVID-19 period, and 74.8% of them were satisfied with e-learning [46].

### Limitations

The current systematic review had several limitations. The high level of methodological and instrumental variations in the selected studies precluded a meta-analysis. Finally, there may have been language bias because only studies in English and Persian were searched.

### Recommendations for future research

Based on the findings of the present systematic review, future research should evaluate the impact of different variables on medical students’ satisfaction with e-learning and examine the advantages and disadvantages of various online teaching approaches. It is also suggested that future studies investigate the limitations and obstacles of e-learning.

### Implications for health managers and policymakers

Given the inevitable rise of online learning and education, it is recommended that health managers and policymakers choose the best online education strategy for medical science students by examining various studies in this area to enhance their satisfaction with e-learning. Additionally, it is recommended that in light of the e-learning process’s recognized flaws and restrictions, an effort be made to address these restrictions in order to enhance the e-learning process and, in turn, increase students’ satisfaction with it.

### Conclusion

According to the above results, approximately half of the 15,473 medical science students in this study were satisfied with e-learning during the COVID-19 pandemic. Factors such as age, gender, clinical year, experience with e-learning before COVID-19, level of study, adaptation content of course materials, interactivity, understanding of the content, active participation of the instructor in the discussion, multimedia use in teaching sessions, adequate time dedicated to the e-learning, stress perception, and convenience had a significant relationship with the satisfaction of medical science students with e-learning during the COVID-19 pandemic. Therefore, due to the inevitability of online education and e-learning, it is suggested that educational managers and policymakers choose the best online education method for medical science students by examining various studies in this field to increase their satisfaction with e-learning.

## Supplementary materials

Supplementary files are available from Harvard Dataverse: https://doi.org/10.7910/DVN/3CEPP1

Supplement 1. Basic characteristics of the included studies in this systematic review.

Supplement 2. Assessment of the quality of the included articles.

Supplement 3. Audio recording of the abstract.
