# Supplementary material for: Medical students’ satisfaction level with e-learning during the COVID-19 pandemic and its related factors: a systematic review
Source: J Educ Eval Health Prof. 2022 Dec 20;19:37. doi: 10.3352/jeehp.2022.19.37 (PMC9899548; doi:10.3352/jeehp.2022.19.37)
Supplement: Supplementary file 1 — Supplement 1. Basic characteristics of the included studies in this systematic review. [file jeehp-19-37-suppl1.docx]

**Supplement 1.** Basic characteristics of the included studies in this systematic review

| First Author/year | Location | Sample size | M/F ratio (%) | Age (yr, mean±SD) | Single/married ratio (%) | Field of study of the participants (%) | E-learning modalities (%) | Platforms used in e-learning (%) | Devices used in e-learning (%) | Previous experience of online classes (%) | Questionnaire | Key results | Axis score |
| --- | --- | --- | --- | --- | --- | --- | --- | --- | --- | --- | --- | --- | --- |
| Abbasi et al. [16] (2020) | Pakistan | 382 | 35.86/64.14 | NA | NA | - Clinical medicine (53.40)  - Dentistry (46.60) | NA | NA | - Mobile (75.65)  - Laptop (21.20)  - Tablet (2.35)  - Desktop computer (0.80) | NA | Researcher-made | - 31.15% of the students were satisfied with e-learning.  - 14.66% of the students preferred e-learning to traditional teaching. | High |
| Al-Balas et al. [13] (2020) | Jordan | 538 | NA | NA | NA | Clinical medicine (100) | - Live streaming (80.86)  - Pre-recorded sessions (11.15)  - Live and pre-recorded (7.99) | - Multiple platforms (64.68)  - ZOOM (35.32) | - Multiple devices (49.63)  - Mobile (35.87)  - Computer (14.50) | NA | Researcher-made | - 26.77% of the students were satisfied with e-learning.  - There was a significant relationship between satisfaction with e-learning and previous enrollment in university curriculum-related e-learning (P<0.015).  - There was a significant relationship between satisfaction with e-learning and the active participation of the instructor in the discussion (P<0.0001).  - There was a significant relationship between satisfaction with e-learning and using multimedia in teaching sessions (P<0.0001)  - There was a significant relationship between satisfaction with e-learning and dedicated adequate time to the e-learning (P<0.0001) | High |
| Amir et al. [17] (2020) | Indonesia | 301 | 14.95/85.05 | NA | NA | Dentistry (100) | NA | - Microsoft Teams  - ZOOM  - Google Meet  - EMAS | NA | NA | Researcher-made | - 38.21% of the students were satisfied with e-learning.  - 44.19% of students preferred e-learning to traditional teaching. | High |
| El Bahloul et al. [23] (2020) | Morocco | 265 | 39.62/60.38 | 19.51 (SD=1.36) | NA | Clinical medicine (100) | - Pre-recorded lectures  - Virtual classes  - Training modules  - PPTs | - YouTube  - Video conferencing platform  - Faulty website  - Teleworking platform  - E-learning website of a university hospital | - Mobile  - Tablet  - Personal computer | NA | Researcher-made | - 66.80% of the students were satisfied with e-learning.  - There was a significant relationship between satisfaction with e-learning and the content of course materials (P<0.05).  - There was a significant relationship between satisfaction with e-learning and interactivity (P<0.05).  - There was a significant relationship between satisfaction with e-learning and understanding the content (P<0.05). | High |
| Fatani [18] (2020) | Saudi Arabia | 662 | 100/0 | NA | NA | Clinical medicine (100) | Web video conference (100) | - ZOOM  - Blackboard Ultra | NA | NA | Reduced SEEQ | - 82.02% of the students were satisfied with e-learning.  - The mean score of the student's overall satisfaction with e-learning was 4.10 (SD=0.88). | High |
| Hameed et al. [24] (2020) | India | 161 | NA | NA | NA | Clinical medicine (100) | - Live online lectures  - Group discussion  - Pre-recorded lectures  - PPTs | - WhatsApp  - Video conferencing platform | NA | NA | Researcher-made | - 52.80% of the students were satisfied with e-learning.  - 56.52% of the students consider live online lectures as the most useful method of online teaching, followed by the use of PPTs (26.70%), discussions in the WhatsApp group (9.31%), and recorded lectures (7.47%).  - 57.76% of the students were satisfied with the frequency of online classes.  - 80.12% of the students were satisfied with the content of online classes.  - 50.93% of the students were dissatisfied with the number of topics presented in online teaching. | Fair |
| Kaur et al. [1] (2020) | India | 983 | 32.61/66.59 | 20.02 (SD=1.45) | NA | - Clinical medicine  - Dentistry  - Other | NA | NA | NA | 12.51 | Researcher-made | - 45.19% of the students were satisfied with e-learning in all aspects.  - 56.66% of the students were satisfied with class materials.  - 31.94% of the students were satisfied with the balance of practical and theoretical knowledge.  - 38.96% of the students were satisfied with the professional development strategy for online training.  - 54.22% of the students were satisfied with the availability of e-resources.  - 44.15% of the students were satisfied with the availability of assistance. | High |
| Kim et al. [25] (2020) | South Korea | 318 | NA | NA | NA | Clinical medicine (100) | - Live online lectures  - Pre-recorded lectures  - Group discussion  - Online virtual slide  - Online simulation program | NA | NA | NA | Researcher-made | - The mean score of students’ satisfaction with e-learning was 3.97 (SD=0.95).  - 62.89% of students preferred online lectures to offline. | High |
| Koirala et al. [26] (2020) | Nepal | 133 | NA | 22.30 (SD=2.90) | 87.97/12.03 | Nursing (100) | NA | ZOOM | - Mobile (51.88)  - Personal computer (24.06)  - Both mobile and personal computer (24.06) | 0 | Researcher-made | 69.17% of the students were satisfied with e-learning. | High |
| Ni et al. [28] (2020) | China | 1,050 | 43.14/56.86 | NA | NA | - Clinical medicine (57.33)  - Basic medicine (6.28)  - Pharmacy (15.14)  - Pediatrics (10.38)  - Medical imageology (8.00)  - Medical laboratory science (2.87) | NA | NA | - Mobile (61.14)  - Tablet (30.57)  - Laptop (71.14)  - Desktop computer (7.24) | NA | Researcher-made | 33.71% of the students were satisfied with e-learning. | High |
| Sindiani et al. [30] (2020) | Jordan | 2,112 | NA | NA | NA | Clinical medicine (100) | - Multiple modalities (45.74)  - Live online lectures (31.44)  - Pre-recorded lectures (22.82) | - ZOOM (63.02)  - Microsoft Teams (10.98)  - Both ZOOM and Microsoft Teams (11.98)  - Others (14.02) | NA | 73.58 | Researcher-made | - 25.00% of the students were satisfied with e-learning.  - 42.00 of the students preferred e-learning to traditional teaching.  - There was a significant relationship between satisfaction with e-learning and experience with e-learning before COVID-19 (P<0.001). | High |
| Vala et al. [33] (2020) | India | 250 | NA | NA | NA | Clinical medicine (100) | Online video lectures (100) | Online video lectures platform (100) | NA | NA | Researcher-made | - 42.80% of the students preferred e-learning classes to traditional teaching.  - 12.00% of the students preferred e-learning for practical classes to traditional teaching.  - The students feel more personalized (55.40%) in the online lectures than in traditional teaching.  - The students have more freedom (68.20%) in online lectures than in traditional teaching. | High |
| Yuryeva et al. [36] (2020) | Ukraine | 203 | 30.05/9.95 | 20.46 (SD=2.79) | NA | Clinical medicine (100) | NA | - Google Classroom  - ZOOM | NA | NA | Researcher-made | - 60.10% of the students were satisfied with e-learning.  - 14.79% of the students preferred e-learning over traditional one. | Low |
| Zhang et al. [14] (2020) | China | 48 | 31.25/93.75 | 21.52 (SD=1.81) | NA | Clinical medicine (100) | Live streaming (100) | Online lectures platform (100) | NA | NA | Researcher-made | - 45.83% of the students preferred e-learning to traditional teaching.  - The mean score of students’ satisfaction regarding the content of e-learning was 3.83 (SD=0.95).  - The results showed that students’ knowledge, behaviors of discussion, and consulting were significantly improved by online courses (P<0.001). | High |
| Alblihed et al. [3] (2021) | Saudi Arabia | 4,850 | 56.60/43.40 | NA | NA | - Clinical medicine (53.61)  - Dentistry (8.66)  - Applied Health (37.73) | NA | - Faculties’ online platforms (75.20)  - Other platforms (24.80) | NA | 77.21 | Researcher-made | - 46.90% of the students were highly satisfied with e-learning.  - Maximum satisfaction was reported among the applied health students (63.13% highly satisfied), followed by dental students (45.00% highly satisfied), and medical students (39.30% highly satisfied).  - The satisfaction with e-learning was significantly higher in females (P=0.000). | High |
| AlQhtani et al. [15] (2021) | Saudi Arabia | 376 | 86.43/13.57 | 22.90 (SD=2.34) | NA | Clinical medicine (100) | - Live online lectures  - Problem-based learning  - Integrated clinical case discussion  - Tutorial  - Self-directed learning | NA | NA | 25.00 | Researcher-made | - 35.88% of the students were satisfied with e-learning in all aspects.  - The mean score of students’ satisfaction was 3.55 (SD=1.20), the highest level, for the availability of e-resources.  - The mean score of students’ satisfaction was 2.07 (SD=1.03), the lowest level, for the practical and theoretical experience.  - The satisfaction with e-learning was significantly higher in males (B=0.38, P=0.001).  - There was a significant negative relationship between satisfaction with e-learning and age (B=-0.05, P<0.05).  - There was a significant positive relationship between satisfaction with e-learning and convenience (r=0.75, P<0.001). | High |
| Dutta et al. [6] (2021) | India | 1,068 | 49.53/50.47 | NA | NA | - Clinical medicine (86.04)  - Nursing (13.96) | NA | NA | - Mobile (81.55)  - Tablet (4.96)  - Laptop (11.70)  - Desktop computer (1.79) | NA | Researcher-made | 37.76% of the students were satisfied with e-learning. | High |
| Li et al. [27] (2021) | China | 230 | 35.65/64.35 | NA | NA | - Clinical medicine (90.00)  - Nursing (10.00) | - Live online lectures  - Pre-recorded lectures  - Group discussions | - Live stream platforms  - Video-uploading websites  - Emails  - Social network software | - Mobile (71.30)  - Tablet (6.95)  - PC (21.75) | NA | Researcher-made | - 36.52% of the students were satisfied with e-learning.  - There was a significant negative relationship between clinical years and satisfaction with e-learning (P<0.05).  - Satisfaction was significantly lower for students inside China (80.50%) than for students outside China (54.90%) (P<0.01). | High |
| Oducado & Estoque [29] (2021) | Philippines | 108 | 29.63/70.37 | 19.91 (SD=0.42) | NA | Nursing (100) | NA | NA | - Mobile (57.41)  - Tablet (2.78)  - Laptop (37.96)  - Desktop computer (1.85) | NA | Researcher-made | - 51.85% of the students were satisfied with e-learning.  - There was a significant negative relationship between stress perception and satisfaction with e-learning (P<0.05). | High |
| Slimani et al. [31] (2021) | Morocco | 154 | 20.78/79.22 | 20.80 (SD=2.80) | NA | Pharmacy (100) | - Pre-recorded lectures  - PPTs | CANVAS (100) | NA | NA | Researcher-made | - 63.64% of the students were satisfied with e-learning.  - 81.82% of the students were satisfied with the structuring of the courses and assessments on the e-learning platform.  - 60.39% of the students were satisfied with the number of topics presented in online teaching.  - 75.32% of the students were satisfied with timetables set for e-learning.  - There was a significant relationship between satisfaction with e-learning and the level of study (P=0.026).  - There was a significant relationship between satisfaction with e-learning and adaptation (P=0.004). | High |
| Totlis et al. [32] (2021) | Greece | 200 | 38.00/62.00 | 20.66 (SD=4.25) | NA | - Clinical medicine (75.50)  - Dentistry (24.50) | - Live online lectures  - Pre-recorded lectures  - PPTs | - University’s online platform  - Skype | NA | NA | Researcher-made | - 56.00% of the students were satisfied with the online anatomy lecture.  - 52.50% of the students were satisfied with pre-recorded anatomy lectures.  - 21.50% of the students were satisfied with studying anatomy lectures’ presentations.  - 49.00% of the students preferred traditional teaching to e-learning methods. | High |
| Vishwanathan et al. [34] (2021) | India | 465 | 50.76/49.24 | 20.00 (SD=1.30) | NA | Clinical medicine (100) | Live online lectures (100) | - Google Classroom  - Google Meet  - ZOOM | - Mobile (86.67)  - Tablet (3.01)  - Laptop (9.67)  - Personal computer (0.65) | NA | Researcher-made | 54.83% of the students were extremely and quite satisfied with e-learning modalities. | High |
| Yekefallah et al. [35] (2021) | Iran | 420 | 26.19/73.18 | 21.14 (SD=1.25) | 53.33/46.67 | NA | NA | NA | NA | 55.47 | Researcher-made | - The mean score of the student's satisfaction with e-learning was 20.75 (SD=2.13).  - 40.95% of the students were satisfied with e-learning.  - There was a significant relationship between satisfaction with e-learning and gender (P<0.05)  - There was a significant relationship between satisfaction with e-learning and previous attending to online classes (P<0.05). | High |
| Yuryeva et al. [19] (2021) | Ukraine | 205 | 30.73/69.27 | NA | NA | Clinical medicine (100) | - Live online lectures  - Pre-recorded lectures | Google Meet (100) | - Mobile (27.31)  - Tablet (0.48)  - Laptop (54.14)  - Desktop computer (16.58)  - Other (1.49) | NA | Researcher-made | - 80.00% of students had moderate and higher satisfaction with e-learning outcomes.  - 15.12% of the students preferred e-learning over traditional ones. | Fair |

M, male; F, female; SD, standard deviation; NA, not available; E-learning, electronic learning; PPTs, PowerPoint files; SEEQ, Students’ Evaluation of Educational Quality; COVID-19, coronavirus disease 2019.
